# Supplementary material for: Fatty Acid Synthase Confers Tamoxifen Resistance to ER+/HER2+ Breast Cancer
Source: Cancers (Basel). 2021 Mar 6;13(5):1132. doi: 10.3390/cancers13051132 (PMC7961649; doi:10.3390/cancers13051132)

Supplementary Materials

Fatty Acid Synthase Confers Tamoxifen Resistance to ER+/HER2+ Breast Cancer

Javier A. Menendez, Adriana Papadimitropoulou, Travis Vander Steen, Elisabet Cuyàs, Bharvi P. Oza-Gajera, Sara Verdura, Ingrid Espinoza, Luciano Vellon, Inderjit Mehmi and Ruth Lupu

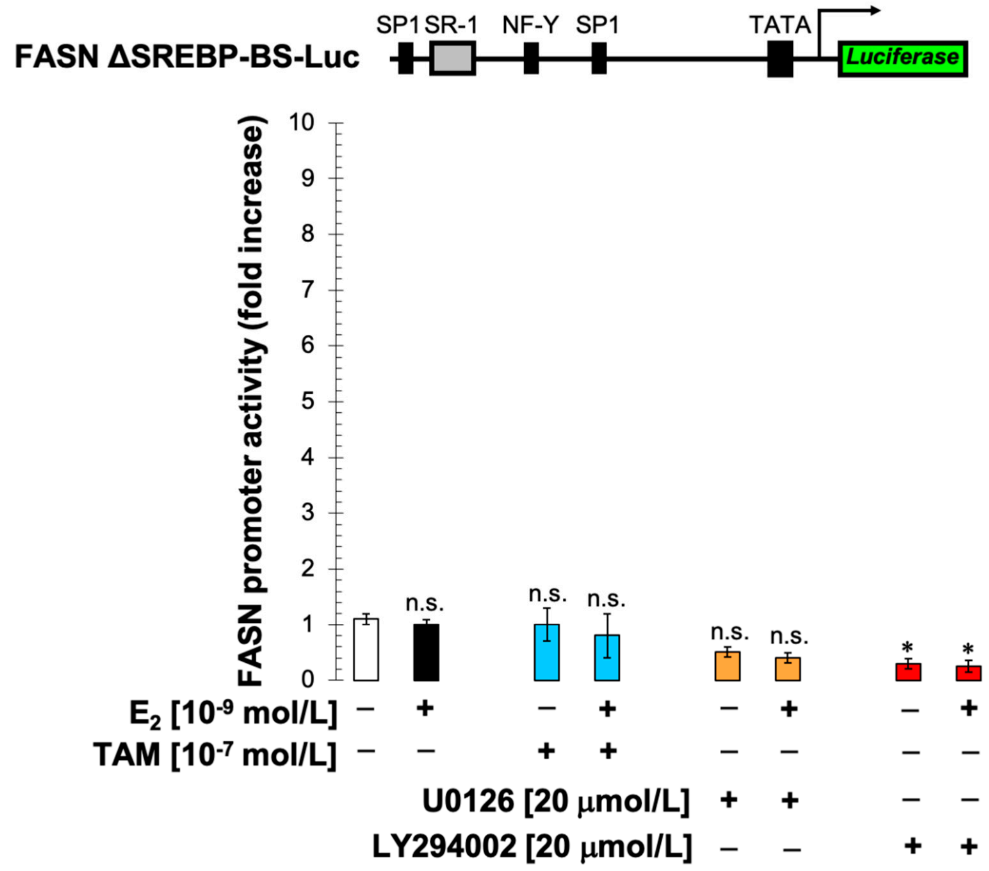

Figure S1. Estradiol/ERα signaling-regulated FASN expression requires a SREBP-binding site at the *FASN* gene promoter. MCF-7 cells were transiently transfected with a plasmid containing a Luciferase reporter gene driven by a 178-bp FASN promoter fragment with the SREBP-binding site deleted and flanked by auxiliary NF-Y and Sp-1 sites. The next day, cells were treated with E_2, tamoxifen, U0126, LY294002 or their combinations, as specified. After 24 h of exposure, cells were lysed and Luciferase activity was measured. Luciferase activity was expressed as relative (*fold*) change in transcriptional activities of FASN promoter-Luciferase-transfected cells in response to treatments after normalization to pRL-CMV activity as described in "Material and methods". Each experimental value represents the mean fold-increase (*columns*) ± S. D. (*bars*) from at least three independent experiments in which triplicate wells were measured. *P < 0.05 and **P < 0.005, statistically significant differences from the untreated (control) group.

Original uncropped gel images **Figure 2A**

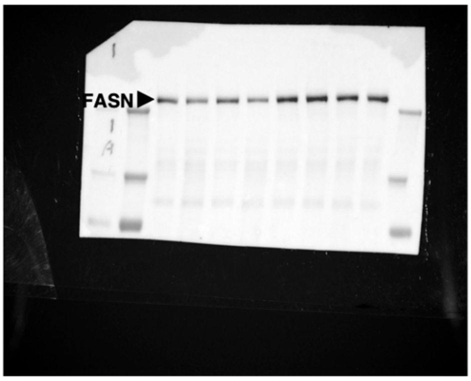

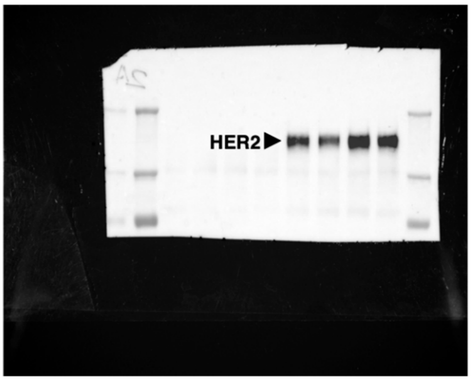

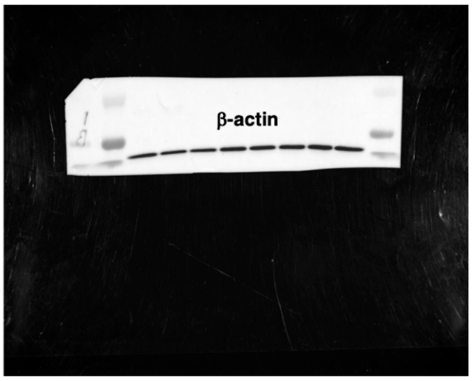

Original uncropped gel images **Figure 3B**

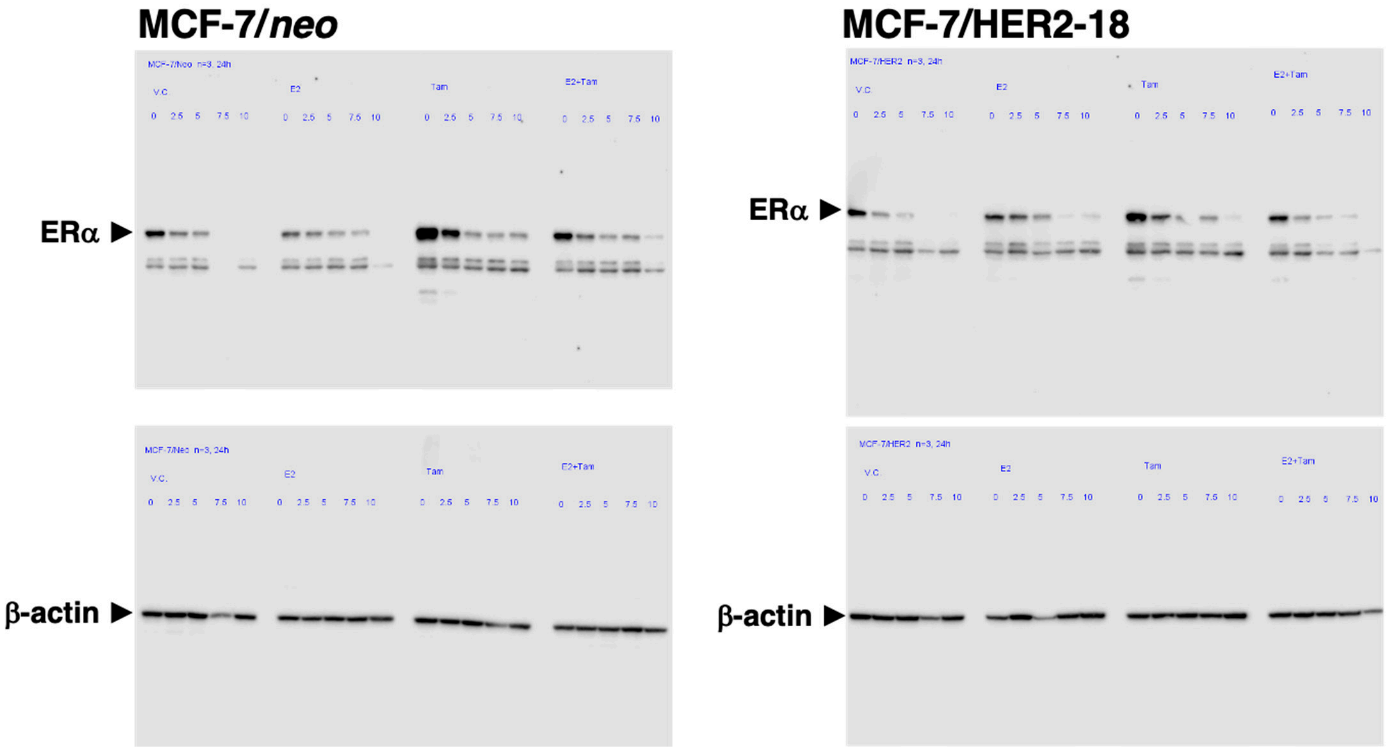

Supplement: Supplementary file 1 [file cancers-13-01132-s001.pdf]
